# The impact of zinc oxide nanoparticles on the bacterial microbiome of activated sludge systems

**DOI:** 10.1038/srep39176

**Published:** 2016-12-14

**Authors:** K. Meli, I. Kamika, J. Keshri, M. N. B. Momba

**Affiliations:** 1 Department of Environmental, Water and Earth Sciences, Faculty of Science, Tshwane University of Technology, Arcadia Campus, Private Bag X680, Pretoria 0001, South Africa

## Abstract

The expected growth in nanomaterial applications could result in increased amounts of nanoparticles entering municipal sewer systems, eventually ending up in wastewater treatment plants and therefore negatively affecting microbial populations and biological nutrient removal. The aim of this study was to ascertain the impact of zinc oxide nanoparticles (nZnO) on the bacterial microbiome of an activated sludge system. A metagenomic approach combined with the latest generation Illumina MiSeq platform and RDP pipeline tools were used to identify and classify the bacterial microbiome of the sludge. Results revealed a drastic decrease in the number of operational taxonomic units (OTUs) from 27 737 recovered in the nZnO-free sample to 23 743, 17 733, and 13 324 OTUs in wastewater samples exposed to various concentrations of nZnO (5, 10 and 100 mg/L nZnO, respectively). These represented 12 phyla, 21 classes, 30 orders, 54 families and 51 genera, completely identified at each taxonomic level in the control samples; 7-15-25-28-20 for wastewater samples exposed to 5 mg/L nZnO; 9-15-24-31-23 for those exposed to 10 mg/L and 7-11-19-26-17 for those exposed 100 mg/L nZnO. A large number of sequences could not be assigned to specific taxa, suggesting a possibility of novel species to be discovered.

Activated sludge refers to a mass of microorganisms which play a crucial role in the biological wastewater treatment process for the production of effluent of high quality prior to its discharge into the receiving water bodies. These microorganisms mainly include bacteria, protozoa, fungi and algae, which are actively involved in the process of removing nutrients and toxic materials from wastewaters^1^,^2^,^3^. Attempts to explain the mechanisms of the nutrient removal process have been focused mainly on dominant bacteria such as nitrifying bacteria, denitrifying bacteria and polyphosphate-accumulating organisms^4^,^5^,^6^. However, these dominant communities have been studied by using the culture-dependent bottom-up or reductionist approach, which does not provide a comprehensive analysis of the microbial communities in wastewater systems^7^. While challenges still remain in isolating and identifying these microorganisms, the disadvantages and inherent biases associated with these culture-based techniques are well documented^8^,^9^,^10^,^11^. It has been reported that over 99% of the microbial communities are not cultivable and results obtained from these methods could lead to a biased representation of certain populations in the samples^7^,^12^. In addition, some bacteria in the activated sludge can remove more than one or even all nutrients from wastewater, but at different rates^13^. The entire nutrient removal process is thus fairly complex because the interactions and possible synergism of environmental organisms are not yet fully understood^14^. Furthermore, factors such as temperature, conductivity, pH, and dissolved oxygen content strongly influence the variance of the bacterial communities in an activated sludge system^15^,^16^,^17^. More importantly, this variance could also be independent of the geographic locations of wastewater treatment plants as well as operating parameters^7^. In recent years, metagenomic approaches have revolutionized microbiology by paving the way for a cultivation-independent assessment approach and exploitation of microbial communities present in complex ecosystems^18^. These approaches are based on direct isolation of nucleic acids from environmental samples and have proven to be powerful tools for comparing and exploring the microbial diversity of environmental samples^19^. Although they are followed by different DNA characterization methods, pyrosequencing has been shown to be an excellent DNA sequencing method, especially for quantitative characterization of microbial communities in wastewater treatment plants^20^,^21^.

Currently, the widespread use of engineered nanomaterials (ENMs) in commercial consumer products and their eventual release into wastewater treatment plants (WWTPs) have raised concern about the impact of these materials on the bacterial activity and the performance of WWTPs. Over 1 600 consumer products have been reported to contain ENMs which sooner or later enter sewer systems and wastewater treatment plants^22^,^23^,^24^. The most frequently used inorganic ENMs in commercial products include nano-TiO_2_, nano-Ag, and nZnO^25^,^26^. Zinc oxide nanoparticles have a wurtzite crystal structure, which contributes to its unique optoelectronic properties^27^. Hence, their various applications and uses in wide-ranging fields such as in food packaging to control food-borne pathogens^28^, in textile industries as surface modifiers for industrial batches of textiles, and as antimicrobials^29^, as well as in laundry additives, clothes and paintings^30^,^31^,^32^. They are also being used extensively in cosmetics, catalysts and in sunblock creams for UV-protection^26^. In wastewater treatment, ENMs adsorb to the activated sludge biomass, and consequently they may negatively impact biological wastewater treatment performance, including nutrient removal^22^. Mu *et al*.^33^ have highlighted the fact that high nZnO concentrations inhibit the activities of protease, acetate kinase and coenzyme F_420_. According to these authors, these enzymes play a key role in the steps of sludge hydrolysis, acidification and methanation during anaerobic digestion of organic substrates, and predominantly, the inhibition of coenzyme F_420_ results in a perturbation in the methanogenic activity of the system^33^. Bajpai *et al*.^34^ pointed out that toxicological studies on nZnO lag far behind the rate at which they are being produced and their increasing number of applications and uses in many fields, and this is due to the traditional concept that nZnO are not toxic. However, the toxic effects of nZnO on different taxa have been confirmed in many studies^35^,^36^, as these nanoparticles can be taken up spontaneously by living tissues such as cell mitochondria and the cell nucleus, resulting in cell membrane alteration, in DNA mutation, in structural damage to mitochondria and eventually in cell death^37^,^38^,^39^. Extensive studies have been carried out on the adverse impact of nZnO on the activities of individual bacteria, particularly on *Escherichia coli* where nZnO led to apoptosis after a series of reactions initiated by the generation of reactive oxygen species (ROS)^36^,^40^. Moreover, studies on the effects of nanoparticles in activated sludge processes are limited to their effects on nutrient removal such as nitrogen and phosphorus removal and biological oxygen demand. In particular, it was found that nZnO toxicity has an adverse effect on the sensitivity of bacterial cultures within WWTPs that remove these nutrients^41^,^42^ or it affects biogas production in anaerobic digestion^43^ without elucidating the impact on the entire bacterial population in terms of abundance and species diversity. To date, the complexity and fate of the ENMs in WWTPs and their adverse impact on the bacterial activity in the activated sludge wastewater treatment plants are not yet well understood, in spite of increasing volumes of ENMs being released into such systems. The present study therefore assessed the impact of zinc oxide nanoparticles (nZnO) on the bacterial microbiome of an activated sludge system, which influences nutrient removal performance.

## Results and Discussion

### Nanomaterial characterization

**Figure 1:**
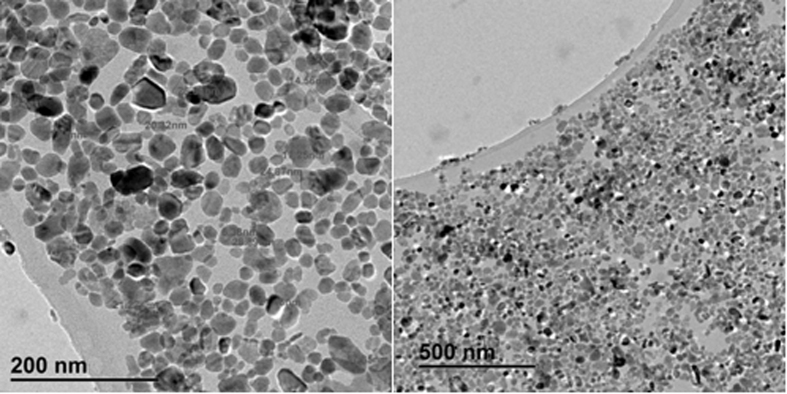
TEM images of nZnO.

The impact of nZnO on microbial populations, untreated activated cells as well as treated cells was microscopically assessed using the transmission electron microscope (TEM). The TEM images have been reported to provide actual size and shape of particles^44^. Figure 1 shows that particles were <100 nm with uneven size and shape (circular, oval, stretched or even irregular shape). This may be the result of the synthesis protocol used to manufacture these nanoparticles since morphological uniformity of nZnO may be obtained by optimising the calcination temperature^45^ or by adjusting the concentration of the ionic template such as potassium nitrate, potassium sulphate or lithium nitrate at different molar ratios^46^ or many other parameters depending on the synthesis protocol used. Particles were found to have a diameter ranging from 6 nm to 60 nm. This observation is consistent with the manufacturer’s information, which reports that particles should be <100 nm. However, information on the size distribution was not provided, neither the dominant size. The TEM images depicted in Fig. 1 show a high congestion of nanoparticles corresponding to the high concentration of dispersed nZnO in water (1.7 × 106 mg/L). For this reason, it would be difficult to ascertain whether the nZnO dispersed in the water were singlets, aggregates or agglomerates based on the classifications by previous investigators^47^,^48^. Further analysis performed from selected areas showed the presence of zinc up to 50 ± 0.35% and the oxygen at 17 ± 0.28% by weight of dried sample on the copper grid, confirming the nature of these nanoparticles being nZnO.

### Diversity indices and community species richness

The outcomes of this study showed a net decrease in the number of metagenomic sequences from all different domains when the nZnO concentration increased (Table 1). It was found that numbers of sequences were reduced by 13.6%, 36.4%; and 52.0% when activated sludge was subjected to the presence of 5 mg/L, 10 mg/L and 100 mg/L nZnO, respectively. However, to ascertain the way in which bacterial communities were affected, the read tags were assigned to different OTUs at 5% nucleotide cut-off using the RDP pipeline. The decision to use the 5% nucleotide cut-off in this study was taken based on the findings of Dalevi *et al*.^49^. In general, a gradual decrease in OTUs was noted as the concentration of nZnO increased in the sample. With a 5% nucleotide cut-off, a total of 27 737 OTUs were recovered from the nZnO-free sample (control), whereas in treated samples, 23 743 OTUs (5 mg/L nZnO), 17 733 OTUs (10 mg/L nZnO) and 13 324 OTUs (100 mg/L nZnO) were recorded. This indicated that the number of OTUs in the activated sludge cultured in samples treated with nZnO was significantly affected by increased nZnO concentrations since p = 0.000650767 (p < 0.05)^50^. The diversity was estimated using the Shannon index (H) and richness estimator Chao1. Similar to OTUs, the Shannon index (H) revealed the impact of nZnO on microbial diversity with the nZnO-free sample (control) having a Shannon index of 10.20133, and the nZnO-treated samples subjected to 5 g/L, 10 g/L and 100 g/L nZnO having a diversity index of 10.04318, 9.76002, and 9.47239, respectively. The constant decrease in the Shannon index when the nZnO concentration increased suggests that bacteria diversity was high and more evenly distributed in the control (nZnO-free sample) than in samples containing nZnO.

The Chao 1 richness estimator was also used to estimate the species richness of nZnO-free and nZnO-treated samples and the analysis revealed that the species richness of samples declined as the nZnO concentration increased (Table 1). A statistical analysis using ANOVA showed that there was a significant difference in community species richness between the control and samples with various concentrations of nZnO (p = 0.001335214). The evenness index (E) was also calculated in order to assess the complexity of individual microbial populations within samples. Unlike the Shannon index, the results showed no difference or only a slight difference between evenness of all samples ranging from 0.99763 to 0.99684. The comparison between number of sequences assigned to the specific taxon and the number of total sequences obtained per sample provided the relative abundance percentage of individual taxa. However, the weakness of this evaluation was evident when underestimated samples or samples that were extremely dissimilar were dealt with, resulting in incoherent values as also previously observed by other investigators^51^.

**Figure 2:**
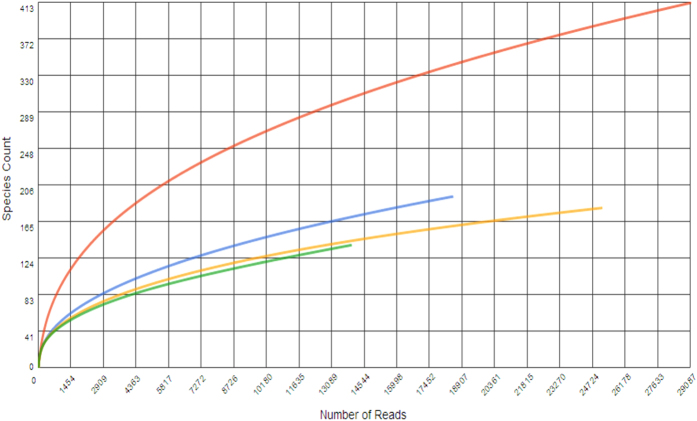
Rarefaction curves of dissimilarity levels calculated for every metagenome illustrating the influence of nZnO: control 0 mg/L nZnO (red), 5 mg/L nZnO (yellow), 10 mg/L nZnO (blue), 100 mg/L nZnO (green). The data were compared to M5NR using a maximum e-value of 1e-5, a minimum identity of 60%, and a minimum alignment length of 15 measured in bp for RNA databases.

The rarefaction curve, which plots the OTU number versus sequence number, was also used to assess the species richness for each individual sample and highlighted the similarity or dissimilarity of their bacterial diversity and community. According to Simberloff^52^, the species richness of an individual sample can be determined as an individual base rarefaction curve that represents the relationship between the numbers of species and OTU numbers at different levels. Figure 2 shows a standardized comparison of species richness for four individual-based rarefaction curves.

Results in the same figure (Fig. 2) also showed the link between the cumulative number of total sequences at any given phylogenetic level (on the x-axis) and the species richness detected in laboratory batch reactor samples (on the y-axis) as also described elsewhere^53^,^54^,^55^. Similar to the Chao 1 richness estimator, the rarefaction curve revealed a decrease in bacterial diversity with increasing nZnO concentrations in the samples. Apart from having a low species richness, nZnO-treated samples revealed a significant dissimilarity (p < 0.05) as compared to controls. Furthermore, a slight plateau on the rarefaction curves for each individual sample (Fig. 2) indicated that a reasonable number of species were recovered and considered. However, a small fraction of the different species still remains to be discovered. This observation was also noted by Schloss *et al*.^56^.

**Figure 3:**
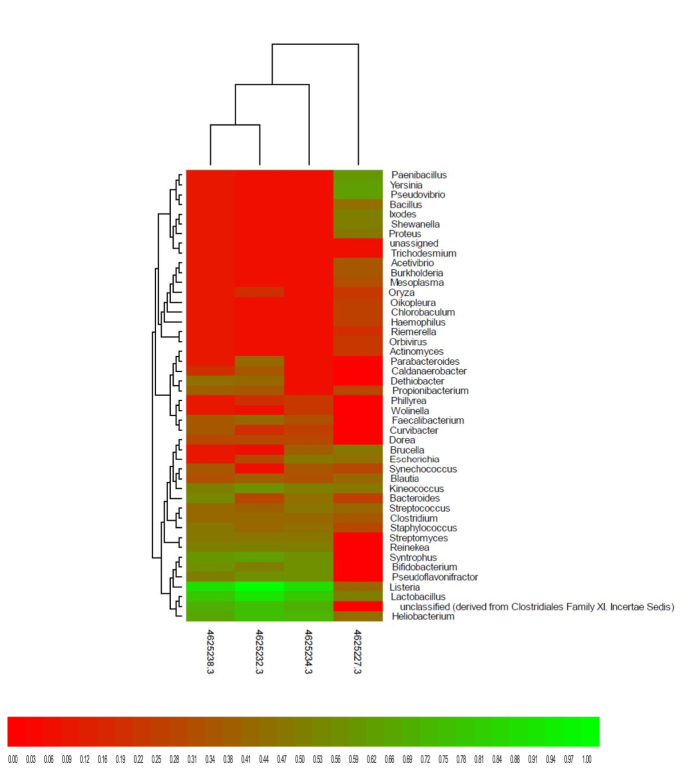
Heat map at genus rank clustering using Ward’s method with Bray-Curtis dissimilarity metric. Legend: 4625227.3 (control), 4625232.3 (5 mg/L), 4625234.3 (10 mg/L), 4625238.3 (100 mg/L).

Further statistical analysis using the Bray-Curtis dissimilarity metric was performed to assess the dissimilarity between samples and also visualised using a heat map. It should be noted that the software used did not have a provision to select a specific domain, for instance bacteria in our study. Therefore, results on the heat map will show sequences from all domains including unclassified groups or non-bacterial sequences (i.e.: *Ixodes*). Results illustrated the relative abundance at genus level in different laboratory batch reactors, revealing the influence of nZnO on the metagenomic profile (Fig. 3). The influence of zinc oxide nanoparticles on bacterial abundance depended on the type of genus; some could not grow even in low concentrations, while other genera only grew in the presence of nZnO. Similar results also showed a drastic change in other genera when grown in the presence of nZnO.

In general, a high bacterial abundance was observed in samples not treated with nZnO (control) as compared to the treated samples. The control samples showed a high proportion ranging from 0.50 to 0.91 for *Paenibacillus, Yersinia, Pseudovibrio, Bacillus, Proteus, Shewanella,* while for the same bacterial genera in treated samples a very low proportion was observed, ranging from 0.00 to 0.09. Similar observations were noted with *Acetivibrio, Burkholderia, Mesoplasma, Oryza, Chlorobaculum, Oikopleura, Haemophilus, Riemerella, Actinomyces,* and *Orbivirus* showing a proportion ranging from 0.32 to 0.50 in the control sample, but 0.00 to 0.09 for treated samples. In contrast, *Parabacteroides, Caldanaerobacter, Dethiobacter, Wolinella, Faecalibacterium, Phillyrea, Dorea, Curvibacter, Reinekea, Streptomyces, Syntrophus, Pseudoflavonifractor,* and *Bifidobacterium,* unclassified (derived from Clostridiales Family XI Incertae Sedis) were found to be unique genera only picked up in the treated samples. Furthermore, *Blautia, Kineococcus, Streptococcus, Clostridium, Staphylococcus, Listeria, Lactobacillus* and *Heliobacterium* were the most abundant genera found in nZnO-treated and untreated (control) samples with samples treated with 5 and 10 mg/L having a very high abundance with a proportion ranging from 0.91 to 1.00 for *Listeria, Lactobacillus* and *Heliobacterium*.

### Fluctuation of bacterial communities in the presence of nZnO

**Figure 4:**
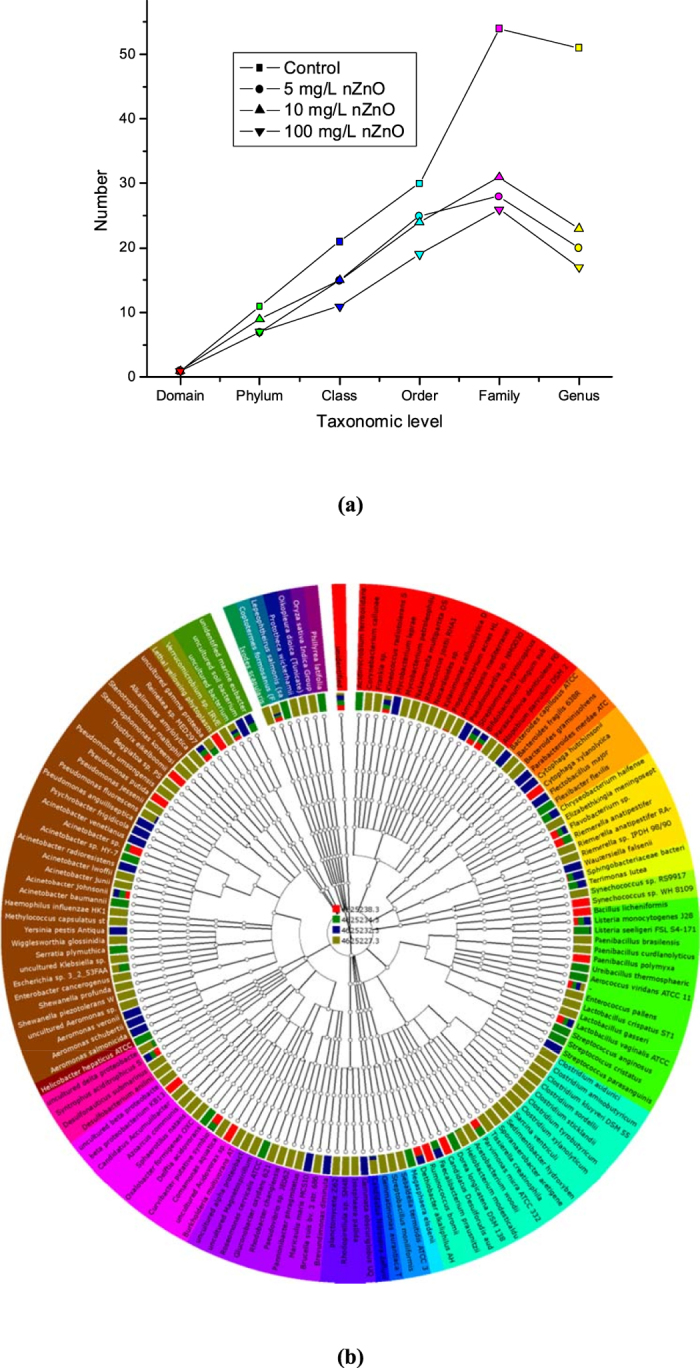
(**a**) Number of bacteria type identified and fully classified per taxonomic rank (excluding all unclassified sequences at each rank). (**b**) Phylogenetic tree of bacterial strains identified in the laboratory batch reactors illustrating the influence of varying concentrations of nZnO: 4625227.3 (control: 0 mg/L), 4625232.3 (5 mg/L), 4625234.3 (10 mg/L), 4625238.3 (100 mg/L).

In the present study, the modified mixed liquor (MML) suggested and prepared according to the protocol described by previous authors^57^,^58^ appeared to support the growth of the bacterial community of the activated sludge in laboratory batch reactors. After 5 days of exposure to various nZnO concentrations in MML as well as in the nZnO-free MML (controls), there were variations in the growth of microorganisms. To comparatively analyze the bacterial communities in the nZnO-free samples and nZnO-treated samples, raw sequences were filtered to remove artefacts; and chimeric sequences were identified and removed using UCHIME as stated elsewhere^59^,^60^. All non-chimeric sequences were assigned to different bacterial taxa as previously described^59^,^60^,^61^. It was also noted that the bacterial community became more diverse as reads were classified into lower taxonomic levels. As can be seen in Fig. 4a, in general, the diversity of the identified bacterial community gradually decreased with increasing nZnO concentrations in the samples, while high numbers of phyla, classes, orders, families and genera were observed in nZnO-free samples. The sample treated with 5 mg/L nZnO revealed 7 phyla, 15 classes, 25 orders, 28 families and 20 genera, while the sample treated with 10 mg/L nZnO showed 9 phyla, 15 classes, 24 orders, 31 families and 23 genera, and in the sample treated with 100 mg/L nZnO 7 phyla, 11 classes, 19 orders, 26 families and 17 genera were observed. These results are also shown in the phylogenetic tree given in Fig. 4b; the sequence tags assigned to the different phylogenetic bacterial taxa also displayed all the changes due to the increase in nZnO. When comparing the effect of nZnO concentrations on the bacterial community, it is important to note that a constant and drastic decrease was prominent only at class and order levels. These results suggest that bacterial communities at these levels may become very sensitive to nZnO at higher concentrations (i.e. from 5 mg/L to 100 mg/L or from 10 mg/L to 100 mg/L).

**Figure 5:**
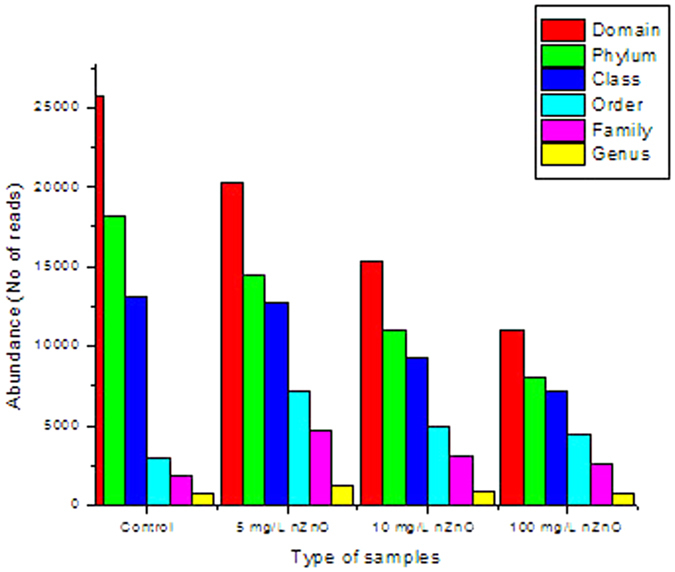
Number of bacterial sequences identified and fully classified per taxonomic rank (excluding all unclassified sequences at each rank).

A net decrease in reads, which indicated a decrease in the abundance of the bacterial domain, coincided with a gradual increase in nZnO concentrations (Fig. 5). The number of reads decreased from 25 730 in the control by 21.06% (20 312 reads); 40.33% (15 352 reads) and 57.09% (11 041 reads) when samples were treated with 5 mg/L, 10 mg/L and 100 mg/L nZnO, respectively. Despite the low variance in the identified number of bacterial phyla found in the treated and untreated samples (11 [control], 7 [5 mg/L nZnO], 9 [10 mg/L nZnO] and 7 [100 mg/L nZnO]), the number of reads at phylum level decreased consistently with increasing nZnO concentrations (18 211; 14 461; 11 042 and 8 083 reads, respectively).

**Figure 6:**
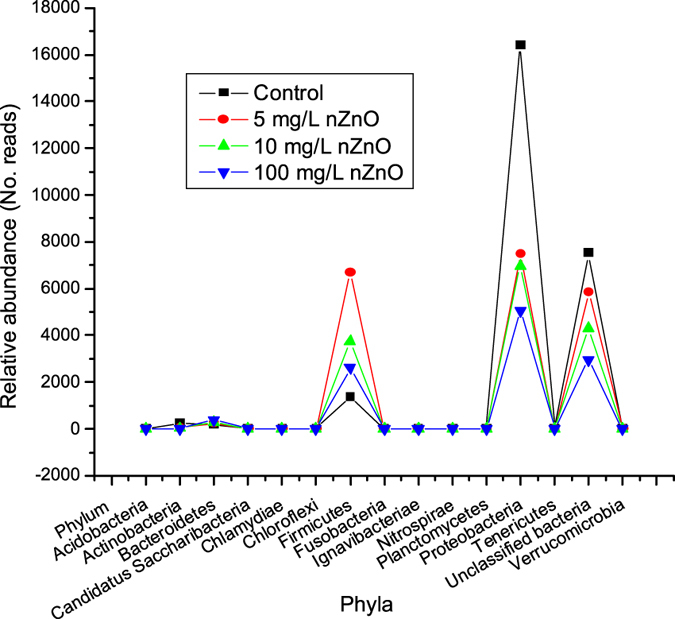
Influence of nZnO concentrations on phylum abundance in wastewater community data obtained at a 95% confidence level.

When considering the abundance of identified bacteria in all treated and untreated samples, as shown in Fig. 6, more than 95% of the total population consisted of Proteobacteria, Firmicutes and unclassified bacteria, with Proteobacteria (63.72% ≈ 16 395 reads, 36.86% ≈ 7 488 reads, 45.28% ≈ 6 952 reads and 45.86% ≈ 5 063 reads for samples treated with 0 mg/L nZnO, 5 mg/L nZnO, 10 mg/L nZnO and 100 mg/L nZnO, respectively) being the predominant phylum. Despite a net decrease due to the increase in nZnO concentrations, they remained the predominant phylum in each sample (Fig. 6). In the control, unclassified bacteria (29.22% ≈ 7 519 reads) was the second most abundant group followed by Firmicutes (5.31%), Actinobacteria, Bacteroidetes, Chloroflexi, Planctomycetes and Fusobacteria (between 0.01 to 0.99%). With the exception of the sample treated with 5 mg/L nZnO, in all treated samples, unclassified bacteria were found to be the second most abundant group (28.07% and 26.79% for samples treated with 10 mg/L nZnO and 100 mg/L nZnO, respectively) followed by Firmicutes (24.45% and 23.66% for samples treated with 10 mg/L nZnO and 100 mg/L nZnO, respectively). In the sample treated with 5 mg/L nZnO, the second most abundant was Firmicutes (32.98%) followed by unclassified bacteria (28.81%). These results revealed a high number of sequences that could not be assigned to specific taxa, suggesting a possibility of an abundance of novel species that are yet to be discovered. The present findings are in agreement with those of Srinandan *et al*.^62^ who also reported Proteobacteria as the most predominant bacterial phylum in the wastewater treatment plant. They also confirmed the findings of Wang *et al*.^63^ who investigated the microbial ecology of 14 wastewater treatment plants across China. Their findings revealed Proteobacteria as the most predominant phylum which constituted approximately 21 to 53% of the bacterial population in the wastewater treatment plants. It is important to note that the three phyla (Bacteroidetes, Acidobacteria, and Chloroflexi) found in the present study were not observed by Wang and his co-workers^63^. These results may suggest that bacterial microbiomes of wastewater systems may vary from one system to another and also from one country to another. Moreover, pH values could also influence the bacterial microbiomes of wastewater systems. In the current study, the pH values were found to be ranging between an initial value of 7.2 and 9.5 at the end of experiments and throughout under aerobic condition. Jones and co-workers^64^ reported that the Acidobacteria phylum is mostly found in acidic and oxygen-depleted environments. This could explain the absence of this phylum in all laboratory batch reactors regardless of the presence of nZnO. When investigating the impacts of nZnO and Ag-NP on functional bacterial community in activated sludge, Chen *et al*.^65^ reported that the phylum Proteobacteria was the most abundant at a relative abundance ranging between 77.8% and 85.4%. However, in the study by Chen *et al*.^65^, only 180 clones were identified and the extraction and sequencing method could not identify unclassified bacteria as in the present study. It should be mentioned that the following phyla - Ignavibacteriae, Tenericutes, and Chlamydiae - were considered as being the most sensitive to nZnO as they were found to be present only in the control. However, in this study the main challenge was the difficulty to compare environmental sample data accurately due to sample size (in a range of microlitres), especially when classifying sequences that were less than 5 reads. This could also explain the higher number of identified bacteria at the family level in the presence of 10 mg/L nZnO than in the presence 5 mg/L nZnO (Fig. 4a). Knowing that these cells had enough time to grow (5 days), it would be erroneous to assume the sensitivity of these cells to nZnO. This observation could be explained either by probable dead cells initially sampled, since metagenomic identification reflects DNA from both live/active and dead cells or by the inability of these cells to grow under operating conditions set in this experimental study series. This could also explain the presence of the phylum Candidatus Saccharibacteria (formerly known as Candidate Division TM7) (1 read) in both untreated and treated samples. However, it will still be necessary to confirm the sensitivity to nZnO at phylum level for Actinobacteria, exclusively gram-positive, which showed a net decrease of 86.80%, 86.80%, and 93.20% when comparing the number of reads in nZnO-treated samples to the number of reads in the control (i.e., 250, 33, 33 and 17 reads for 0, 5, 10 and 100 mg/L nZnO, respectively). The same scenario could be observed at class level with the sole bacteria being Actinobacteria.

The Bacteroidetes phylum, exclusively gram-negative, showed a particularity of having reads increase together with an increase in nZnO concentration, especially at class level with Flavobacteria (i.e., 26, 167, 237 and 345 reads for 0, 5, 10 and 100 mg/L nZnO, respectively), suggesting that they were not affected by the presence of nZnO. These results are in line with those of previous studies which suggest that gram-negative bacteria are more resistant to nanoparticles due to their multilayered cell membrane structure which is composed of lipopolysaccharides, phospholipids, protein molecules, and surrounded by a thin peptidoglycan cell wall. Indeed, these layers form a protective barrier, preventing damage to the bacterial cell^37^,^66^,^67^. The proliferation of these bacteria in treated samples occurred under conditions that are less conducive to bacterial growth and where a decrease in more sensitive bacteria would be expected. The only class that seemed to be affected by nZnO concentration was Bacteroidia (i.e., 64, 1, 1 and 2 reads for 0, 5, 10 and 100 mg/L nZnO, respectively).

When considering the prevalence of Proteobacteria at class level, in all laboratory batch reactors, the Gammaproteobacteria were found to be largely dominant compared to other proteobacterial classes. For example, Proteobacteria in the control were constituted mainly of Gammaproteobacteria (60.82%), and together with the unclassified Proteobacteria (29.62%), they made up more than 90% of the Proteobacteria observed. The same trend could be observed in samples treated with varying concentrations of nZnO. When analysing the impact of nZnO, it was observed that the reads of the proteobacterial classes decreased drastically with the increase in nZnO concentrations. Gammaproteobacteria (9 971 reads in control) decreased by 43.39% (5 645 reads), 53.97% (4 590 reads) and 70.27% (2 964 reads) when treated with 5, 10 and 100 mg/L nZnO, respectively. These results are in line with those of previous studies on isolated bacteria belonging to the group of *γ-Proteobacteria* such as *Pseudomonas aeruginosa*^37^,^68^, *Vibrio fischeri*^69^, *Escherichia coli*^37^,^70^. These bacteria showed high sensitivity to nZnO, predicting that bacteria belonging in this group can be affected by nZnO. Alphaproteobacteria (944 reads in control) decreased by 88.88% (105 reads), 86.23% (130 reads) and 94.81% (49 reads) when treated with 5, 10 and 100 mg/L nZnO, respectively. However, Betaproteobacteria were not affected by the presence of nZnO; they were even found to increase with increasing nZnO concentrations (i.e., 616, 676, 889 and 1 398 reads, respectively, in control, 5, 10 and 100 mg/L nZnO), more specifically the Burkholderiales order.

At lower classification level of the phylum Firmicutes, class Bacilli was found to be extremely dominant at more than 90% of Firmicutes classes in samples containing 5, 10 and 100 mg/L nZnO, while the control was dominated by class Clostridia which was absent in samples treated with nZnO, revealing the high sensitivity of Clostridia to nZnO. The inhibition of species belonging to the class Clostridia was seen as an alarming situation since these species are involved in the fermentation of organic matter to produce volatile fatty acids needed for the removal of phosphorus^71^. In the studies of Cydzik-Kwiatkowska and Zielińska^72^ it was noted that *Clostridium* and *Bacillus* species are common and an important part of the full-scale wastewater treatment systems.

In addition, the present study revealed unique species at different taxonomic ranks in treated samples. It can be observed, specifically for samples treated with 100 mg/L nZnO that species uniqueness appeared only at family and genus levels. Remarkably, all unique reads in treated samples were found to be less than 6 reads, suggesting either that they could not grow in such environment or that the DNA of dead cells was picked up. In contrast, in the untreated sample, at genus level, 55 unique genera observed (542 reads = 2.12% of total bacteria) had an abundance ranging from 137 reads to 1 read. Among the core unique genera described, most abundant unique unclassified-genera (Proteiniclasticum, Proteocatella, unclassified Clostridiales-Incertae Sedis XII) belonging to Clostridiales as well as Rhodobacteraceae (unique genus *Gemmobacter*) families were identified in the control samples and they have been reported to be involved in the fermentation process in wastewater treatment^73^,^74^. Further unique unclassified genera (30 reads) belonging to Porphyromonadaceae family were observed; they are known to form part of the human intestinal tract community^75^. Urban wastewater is a combination of domestic and industrial wastewater streams and human gut bacteria are thus part of the core families found in urban wastewater inflows into WWTPs^76^. In addition, unique genera (the genus *Insolitispirillum*, unclassified Neisseriaceae, unclassified Rhodospirillaceae) belonging to nitrifying bacteria were also identified in the control sample^77^. The genus unclassified Peptostreptococcaceae observed in the present study as unique genus for control samples has also been found in several wastewater treatment systems but no details on contaminant removal have been reported^77^. With such a large number of unique reads in the control, it can be suggested that these organisms were extremely sensitive and incapable of growing in the presence of nZnO and this in turn can disrupt the performance of the wastewater treatment system^78^. This dissimilarity was confirmed using the Jaccard index that showed high dissimilarity among treated and untreated samples at values ranging between 0.91 and 0.98 with the pair control and the 100 mg/L nZnO sample showing the highest dissimilarity (0.98).

### Physicochemical parameter changes during experiments

Table 2 summarizes the changes in physicochemical parameters in the untreated and treated samples. In general, an increase in the values of physicochemical variables was observed except for dissolved oxygen (DO) that showed a decrease throughout the experimental period. Furthermore, nZnO concentrations did not show a significant effect on physicochemical variables as the DO concentrations did not change subsequently with a gradual increase in nZnO concentrations. Most of the samples appeared to vary from almost neutral pH to highly alkaline (approximate value: pH 9.76) after 5 days of incubation. Concentrations of DO revealed a decrease of approximately 30% in all samples (treated and untreated).

**Figure 7:**
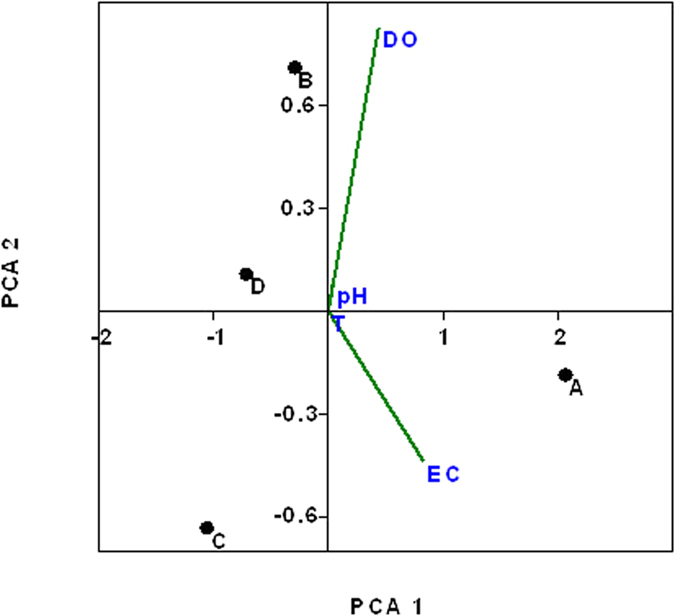
Principal component analysis (PCA) of physicochemical parameter changes during experiments.

Despite the impact of zinc oxide nanoparticles on the bacterial microbiome in the present study, the physicochemical parameters were reported to play a major role in microbial diversity in activated sludge, and to establish the relationship between them and microbial composition in individual samples, a principal component analysis (PCA) was performed (Fig. 7). Beside the fact that a strong relationship was observed between conductivity and dissolved oxygen, the control sample revealed higher conductivity values than the treated samples. The relationship between EC and DO was hypothesized by the production of extracellular compounds coupled with the bacterial growth leading to the consumption of oxygen in the media. However, in the treated samples, the conductivity appeared to decrease due to the toxicity of nZnO on bacterial species inhibiting their growth and later the production of extracellular compounds. Similarly, the DO value was high in the control and lower in all treated samples (Table 2). However, the temperature was maintained constant at 35 ± 2 ^o^C during the study period and a similar pH value was recorded in all samples, highlighting that these parameters did not have a major influence on microbial dissimilarity but instead played the main role in microbial similarity. The results of the present study are in agreement with the findings reported elsewhere showing that electrical conductivity (EC) is a significant parameter regulating microbial composition and diversity in a particular ecosystem^63^,^79^,^80^. Yang *et al*.^15^ also reported that conductivity values are one of the main parameters that show a strong correlation with bacterial growth. According to Fierer *et al*.^81^, temperature, pH and DO are also important factors as they regulate the overall bacterial diversity and composition in the ecosystem. However, the findings of this study disagree with those of Siggins *et al*.^82^, as it was revealed that temperature and pH did not play a significant role in microbial dissimilarity in both nZnO-treated and untreated samples.

### Morphological changes and nanoparticle interaction with cells

**Figure 8:**
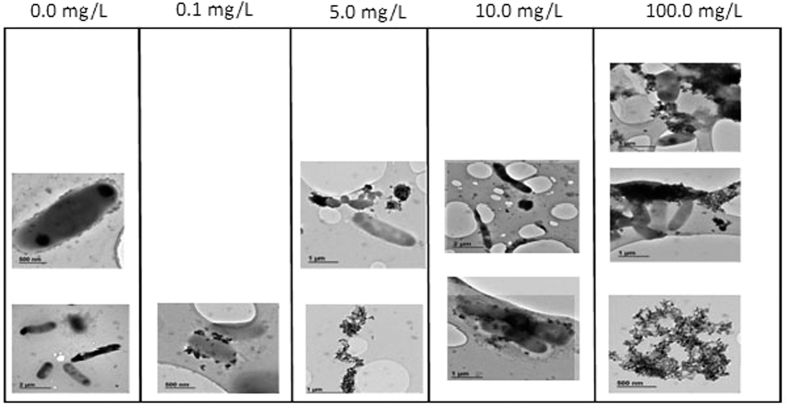
TEM images of nZnO effects on sludge culture.

In order to further monitor the impact of nZnO on the microbial population, the morphological changes of the community as well as their interaction with nZnO were microscopically assessed using TEM. Results obtained showed different interactions with nZnO depending on the type of microorganisms (Fig. 8). At lower concentrations, nZnO particles were observed to first attach to the bacterial cells before damaging the cells as the concentrations increased. The size-dependent effects of nanoparticles on cells have been reported; small particles are more toxic than larger particles^83^. In this study, the toxicity of nZnO particles was mostly due to agglomeration/aggregation as most of the particles could not penetrate inside the cells due to the lack of homogeneity in particles sizes that varied from 6 to 60 nm (Figs 1 and 8). Yamamoto^84^ and Jones *et al*.^85^ in their studies determined different modes of action on isolated bacterial cells which differ as well depending on the type of cells. It has been reported that nanoparticles can alter cell membranes leading to cell death^86^. This was revealed in samples treated with high nZnO concentrations and findings are in agreement with those of Yin and Casey^87^ who stated that high concentrations of nZnO can easily alter cell membranes due to the high level of reactive oxygen species (ROS) produced. These groups of free radicals are produced through a sequential reduction of oxygen by electron addition to the oxygen molecule during mitochondrial electron transport^88^,^89^. This can explain the depletion of dissolved oxygen since the increase of stress due to the increase in nZnO concentration leads to the production of extracellular compounds and consumption of DO. A number of investigators^90^,^91^,^92^ have pointed out that that during the production of ROS, which is harmful to cells, a natural defensive mechanism is initiated that involves enzymatic antioxidants, water or fat-soluble non-enzymatic antioxidants. When excessive ROS are produced, the defence limit can be reached, disrupting the cell membrane and then leading to cell death. However, other studies have revealed that some microbial species can survive in the environment containing nZnO as the nanoparticles interact differently, depending on whether they are gram-negative or gram-positive cells^37^.

## Conclusion

For the first time, the Illumina next-generation sequencing platform was used to reveal the impact of zinc oxide nanoparticles on the microbiome profile of activated sludge systems including cultivable, uncultivable as well as unclassified bacteria. Based on the Shannon diversity index, the microbiome observed in the current study revealed that bacterial diversity and distribution are considerably affected by the presence of nZnO in laboratory batch reactors. The decrease was also confirmed by the Chao 1 species richness estimator as well as the rarefaction analysis which showed a good sampling of all the recoverable species.

## Methods and Materials

### Sampling of Activated sludge effluent and preparation of modified mixed liquor media

Raw wastewater was collected from the Daspoort Wastewater Treatment Plant (DWWTP) at the outlet of the primary zone, using a sterile 20 L container. The wastewater was allowed to settle for two hours, and then filtered using Whatman No. 1 filter papers to remove biomass and other suspended solids. The filtrate was used to prepare the culture media (modified mixed liquor (MML)) by adding carbon sources and nutrient supplements as follows: 5 g/L sodium acetate; 2.5 g/L d-glucose anhydrous; 0.5 g/L MgSO_4_•7H_2_O and 0.18 g/L KNO_3_. The pH was adjusted at 7.2 ± 0.3 using 1.0 M HCl and 1.0 M NaOH, and finally the modified mixed liquors were sterilized at 121 °C and at a pressure of 200 kPa for 15 min in an autoclave prior to use in any of the experiments conducted.

Prior to use, a suspension of the test nanoparticles (nZnO) purchased from Sigma Aldrich (Sigma, Germany), was prepared with sterile Milli-Q water at a concentration of 1 000 mg/L. From this solution, aliquots of specific volume corresponding to the final nZnO concentration of the working solution (0 mg/L, 5 mg/L, 10 mg/L and 100 mg/L) were added to each sterile 250 mL flask containing the culture medium to obtain a final volume of 100 mL. The return activated sludge samples of the same wastewater treatment plant were collected during the winter season in 2014 and used as the source of the microbial population (inoculum). Activated sludge samples were collected in sterile plastic containers (1 L) and were stored in the fridge at ±2 ^o^C until use. The study was conducted in duplicate for each laboratory batch reactor.

### Characterisation of zinc oxide nanoparticles (nZnO)

Zinc oxide, dispersion nanoparticles, <100 nm particle size, were purchased from Sigma-Aldrich (Switzerland). Prior to use, zinc oxide nanoparticles were characterized in terms of morphology, size of particle and chemical composition using a transmission electron microscope (Model JEM-2100F (HR), JEOL Ltd., Japan) operating at an accelerating voltage of 200 kV.

### Nanoparticle stress experimental study

In order to assess the impact of zinc oxide nanoparticles on the microbial community of wastewater treatment plants, the culture media (treated and non-treated mixed liquor) were inoculated with a 1 mL aliquot of well-mixed activated sludge. The non-treated mixed liquor which contained the mixed liquor medium without nZnO was used as control. Experiments were performed at 35 ± 2 ^o^C on a shaking incubator at 120 r/min for 96 h under aerobic conditions. Aliquots were taken at the start of the experiment and at the end of the incubation period (96 h) to analyse physicochemical parameters such as pH, electric conductivity (EC) and dissolved oxygen as well as microbial compositions. Physicochemical parameters were measured using the HQ40d portable pH, Conductivity, Dissolved Oxygen, ORP, and ISE Multi-Parameter Meter (HACH, Germany). To monitor the impact of nZnO on microbial populations, untreated activated cells as well as treated cells were microscopically assessed using the transmission electron microscope (TEM) model JEM-2100F (HR) JEOL operating at an accelerating voltage of 200 kV. After 5 days of incubation, a drop of inoculum was dried on the copper grid used as support to observe the physical impact of nZnO on communities as well as their interaction with nZnO.

### DNA extraction, PCR amplification and sequencing

Genetic material of microbial communities was recovered directly from the treated and non-treated mixed liquor at the initial and final incubation period by centrifuging 50 mL of samples and cleaned twice using sterile PBS X1. Cell pellets were re-suspended in 1 × TE buffer (pH 8.0), then mixed well to create a homogeneous suspension of cells and bacterial DNA was extracted using the ZR Fungal/Bacterial DNA MiniPrep Kit (Zymo Research, Pretoria, South Africa) according to the instructions provided by the manufacturer. Thereafter, the integrity and purity of the eluted bacterial DNA were assessed on the 0.8% agarose gel and measured using a Nanodrop spectrophotometer (Nanodrop 2000, Thermo Scientific, Japan). Universal primer pairs were used for PCR amplified gDNA samples targeting the 16 S rRNA gene (including the primer pair 341F-785R - targeting V3 and V4 regions of the rRNA gene)^93^. Each PCR reaction mixture of 50 μL contained Dream Taq™ PCR master mix (10 × Dream Taq™ buffer, 2 μM dNTP mix and 1.25 U Dream Taq™ polymerase) (2X) (25 μL), nuclease-free water (21 μL), forward primer (1 μL) (0.2 μM) and reverse primer (1 μL) (0.2 μM), and 2 μL of DNA template (50 ng/μL). The following PCR cycling parameters were used: initial denaturation step at 95 °C for 5 min, followed by 30 cycles comprising of denaturation at 95 °C for 40 s, annealing at 55 °C for 2 min and extension at 72 °C for 1 min, with a final extension step for 10 min at 72 °C. The PCR reaction of the re-amplification was as reported previously, but with a slight modification on annealing temperature at 50 °C^ ^94. Based on library concentrations and calculated amplicon sizes, the triplicate samples were pooled in equimolar concentrations. The pooled PCR products were then sequenced at Inqaba Biotechnology Industries, South Africa on the Illumina next generation sequencing platform, using a MiSeq v3 (600 cycles) kit, then 20 Mb of data (2 × 300 bp long paired-end reads) were generated for each sample.

### Sequence analysis

Prior to use, raw sequences were processed for quality by removing sequencing artefacts from the original data sets^95^. Sequences of good quality were further processed for the removal of chimeric reads using UCHIME according to the *de novo* method^96^. The non-chimeric reads were later used for further analysis using the Ribosomal Database Project (RDP) pipeline at a confidence threshold of 95% for microbial classification, and genetic distance was also determined. Reads with a similarity of more than 97% were clustered within the same operational taxonomic unit (OTU) and rarefaction curves were also determined^59^,^61^. Genetic distance was determined and sequences clustered into operational taxonomic units (OTUs) at 0.05% genetic distance using the nearest neighbour method, and a representative sequence chosen for each cluster/OTU.

### Statistical analysis

The Shannon diversity index (H) and the Chao 1 species richness estimator were determined to estimate the microbial diversity and richness in each water sample. The relative abundance (%) of individual taxa within each community was calculated by comparing the number of sequences assigned to a specific taxon against the number of total sequences obtained for that sample. The similarity and dissimilarity in bacterial community structure within the control free of nZnO and all nZnO-treated laboratory batch reactors were analysed using the Jaccard index^59^ and the Bray-Curtis index^97^. This information was used to assess microbial fluctuation by comparing untreated and treated samples. Finally, the analysis of variance (ANOVA) test (Microsoft Excel, Windows 8) was used to analyse the variance induced by nZnO on the microbiome in activated sludge.

## Additional Information

**How to cite this article:** Meli, K. *et al*. The impact of zinc oxide nanoparticles on the bacterial microbiome of activated sludge systems. *Sci. Rep.* **6**, 39176; doi: 10.1038/srep39176 (2016).

**Publisher’s note:** Springer Nature remains neutral with regard to jurisdictional claims in published maps and institutional affiliations.

## Figures and Tables

**Table 1:** Diversity indices of activated sludge metagenome influenced by nZnO.

Sample ID	No. of sequences	OTU	Chao 1	Shannon	Evenness index (E)
Positive control	29 103	27 737	554 104.06442	10.20133	0.99715
S_5 mg/L nZnO	25 139	23 743	356 974.84407	10.04318	0.99684
S_10 mg/L nZnO	18 504	17 733	344 286.66519	9.76002	0.99763
S_100 mg/L nZnO	13 963	13 324	236 445.14865	9.47239	0.99738

**Table 2:** Physicochemical parameter changes during experiments.

nZnO concentration	DO (mg/L)		Conductivity (ms/cm)		pH	
	Initial	Final	Initial	Final	Initial	Final
0 mg/L	9.94	7.13	5.25	8.565	7.2	9.76
5 mg/L	9.94	6.82	5.25	6.06	7.2	9.77
10 mg/L	9.94	5.28	5.25	6.01	7.2	9.62
100 mg/L	9.94	6.1	5.25	5.97	7.2	9.63
